# Effects of 12-week combined exercise on RANKL/RANK/OPG signaling and bone-resorption cytokines in healthy college females 

**DOI:** 10.20463/jenb.2019.0003

**Published:** 2019-03-31

**Authors:** Ji-yeon Kim, Hyo-Jin Kim, Chang-Sun Kim

**Affiliations:** 1 Department of Counseling, Health & Kinesiology, Texas A&M University-San Antonio, San Antonio USA; 2 Department of Physical Education, Dongduk Women's University, Seoul Republic of Korea

**Keywords:** RANKL, RANK, OPG, combined exercise, Healthy college females

## Abstract

**[Purpose]:**

The OPG/RANK/RANKL signaling is a new family of bone metabolism biomarkers belonging to the immune system. However, the bone metabolism response to long-term exercise in the RANKL/RANK/OPG signaling is less evident. The purpose of this study was to examine these biomarkers in healthy college females after 12-weeks combined exercise intervention.

**[Methods]:**

Participants (N=22, 22.4±1.3yrs) were randomly divided in two different group: 12 in the control group and 10 in the exercise group performing combined exercise program that interventions was conducted 3 times per week for 12 weeks. The outcome measures included serum concentrations of RANKL, OPG and bone metabolic cytokines such as TNF-α and IL-6, and mRNA expressions of same variables from PBMC. VO_2max_ and bone mineral density (BMD) were measured at before and after exercise intervention.

**[Results]:**

There were no significant differences in the serum RANKL, OPG concentrations and all RANKL/RANK/OPG signaling mRNA expression on interaction effect between group and time (NS). Also no significant differences were found in the serum TNF-α and IL-6 concentrations and mRNA expression (NS). The IL-6 mRNA expression only showed significant difference in the main effect of groups (p<.05). There were also no significant differences in the VO_2max_ and BMD on interaction effect between group and time (NS).

**[Conclusion]:**

These results suggested that there were no effects on bone mineral density and RANKL/RANK/OPG signaling without the effect of 8-weeks combined exercise on cardiovascular endurance fitness.

## INTRODUCTION

Bone mineral density is influenced by exercise, nutrition, and other lifestyle factors, and bone metabolism is modulated by the interaction between bone resorption from osteoclasts and osteogenesis from osteoblasts. Physical strain, such as that from body weight, greatly affects the bones^1^, and it is demonstrated that regular and adequate exercise improves bone mineral density^2^. The effect of exercise likely depends on the form and intensity of the exercise as well as time, frequency, and other conditions. In addition, the effect of exercise on increasing bone mass is known to be caused by accelerated osteogenesis^3^ and inhibition of bone resorption^4^, but its specific mechanism is still unclear. Recent studies have shown a lack of evidence supporting the inhibition of bone resorption upon exercising.

The pro-inflammatory cytokines, including interleukin 6 (IL-6)^5^ and tumor necrosis factor–alpha (TNF-α)^6^, directly stimulate osteoclast differentiation, proliferation, and activation and are known to promote bone resorption^7^^,^^8^^,^^9^. Moreover, the recently identified receptor activator of nuclear factor kappa-B ligand (RANKL) is an important cytokine that sustains osteoclast formation and survival^10^. The binding of RANKL and its receptor, RANK, triggers the activation of signaling related to the formation and differentiation of osteoclasts and regulates bone resorption^11^. Additionally, osteoprotegerin (OPG) acts as a decoy receptor that binds to RANKL, thereby playing a role in blocking osteoclast differentiation and activation^12^, which is called RANKL/RANK/OPG signaling. It is useful to clarify the resorptive activity of osteoclasts through examining changes in expression of RANKL/RANK/OPG signaling components. However, most previous studies on the RANKL signaling factors focused only on pharmaceutical research for patients with osteoporosis^13^ and the effect of exercise is scarcely discussed. Previous studies have revealed that exercise generates inflammation factors as in the case of TNF-a and IL-1b^7^; it is thought that exercise influences osteoclast metabolism, but evidence remains insufficient.

We have conducted several studies to investigate the effect of exercise training on bone-resorbing cytokines and RANKL signaling factors. To evaluate the effect of acute exercise on bone-resorbing cytokines in young men, the subjects performed acute exercises at 70%, 80%, 90%, and 100% of their ventilation thresholds (VT)^14^. As a result, the expression of bone-resorbing cytokines did not statistically significantly differ among groups subjected to different acute exercise intensities. Our previous study results showed that TNF-α and IL-6 mRNA expression from peripheral blood mononuclear cells (PBMCs) was significantly elevated in elderly women with osteopenia after completing 60 min of acute pilates exercises^15^. In addition, we investigated the effect of acute cycle ergometer training (60% VO_2max_) on bone-resorbing cytokines in elderly women with osteopenia and found that concentrations of serum and mRNA expression of bone-resorbing cytokines from PBMCs were not significantly altered^16^. We also elucidated the effects of high- (80% VO_2max_) and low-intensity (40% VO_2max_) acute cycle ergometer exercise. As a result, the concentration of serum OPG was increased, but no change was observed in levels of the other bone metabolic cytokines after high-intensity exercise in elderly osteoporotic women^17^. Furthermore, serum concentrations of RANKL and OPG and the mRNA expression of RANK, RANKL, and OPG were not significantly changed upon acute treadmill exercise training reaching 60 or 80% VO2max in healthy college female subjects^18^. In contrast, a study reported the effect of eight weeks of pilates exercise on bone metabolic cytokines in female college students; IL-6 and TNF-α mRNA expression was not significantly changed^19^. Despite ongoing research efforts, a consistent effect of acute exercise with various intensities or prolonged exercise on bone-resorptive cytokines or RANKL signaling factors has yet to be observed. Further studies are needed to understand the precise mechanisms underpinning these pathways.

Therefore, the purpose of this study was to examine the effect of a 12-week combined exercise program on the RANKL/RANK/OPG signaling and bone resorption cytokines in healthy college females. In this study, combined exercise, which is known to have a positive effect on bone metabolism, was chosen as the exercise method.

## METHODS

### Research subjects and procedures

The subjects, who voluntarily participated, were recruited from D university (22.41 ± 1.30 y). Twenty-four subjects who had not been regularly physically active during the previous six months were selected (Table 1). Two subjects were excluded due to under-attendance (attendance rate 80% or less) and finally, participants were assigned into the combined exercise group (EXE; n = 10) and the control group (CON; n = 12). The baseline measurement before the experiment found no difference between the two groups. At the beginning and end of the experiment, the subjects underwent body composition measurement, bone mineral density analysis, a maximum oxygen uptake (VO_2max_) test, and blood collection. The CON group controlled their medication, diet, and regular exercise that could affect results during the 12-week study period. The EXE group performed a combined exercise program three times a week, for 60 minutes a session, for 12 weeks. After 12 weeks, the post-test measurement was taken using the same methods as before. This study was processed with the approval of the Bioethics Committee of the public Institution designated by the Ministry of Health and Welfare (National Bioethics Committee, P01-201511-13-001).

**Table 1:** Characteristics of study subjects

Variables	Group	Before	After	Source	*F*	*P*
Weight (kg)	CON EXE	54.3 ± 4.0 53.9 ± 6.6	54.4 ± 4.7 52.9 ± 5.9	group time g×t	0.043 4.086 1.801	0.84 0.074 0.213
muscle mass (kg)	CON EXE	34.5 ± 2.2 34.0 ± 3.0	34.3 ± 2.4 34.2 ± 3.2	group time g×t	0.208 0.128 0.925	0.659 0.729 0.361
Body fat (%)	CON EXE	32.2 ± 6.9 32.1 ± 7.0	33.6 ± 6.8 31.3 ± 6.1	group time g×t	0 0.102 2.767	0.999 0.757 0.131

Mean ± SD. CON, Control group; EXE, Exercise group; BMI, Body Mass Index; g × t, group × time; ^*^:p < 0.05 vs before.

### Combined exercise program

For the EXE group, a combined exercise program that consisted of 30 minutes of endurance exercise and 30 minutes of resistance exercise was applied. The load intensity was gradually increased every four weeks (Table 2). The VO_2max_ value was used to calculate the endurance load intensity. The treadmill running speed was calculated as follows: 1-4 weeks (50% VO_2max_), 5-8 weeks (60% VO_2max_), 9-12 weeks (70% VO_2max_). The resistive load intensity was measured by 1RM (repetition maximum) for each muscle part using weight training machine including chest press, lat pull down, shoulder press, biceps curl, triceps extension, abdominal, leg press, leg extension, and leg curl. 1RM was determined through the indirect method [1RM = load weight × (1 + 0.025 × number of repetitions)] described by O'Conner^20^. For each muscle part, two sets of eight to 12 repetitions were performed. The exercise load intensity was gradually increased as follows: one to four weeks (60% of 1RM), five to eight weeks (70% of 1RM), 9-12 weeks (85% of 1RM).

**Table 2:** Endurance and resistance training program

Endurance training		VO_2max_ (ml/kg/min)	Running speed (mph)		
			50% VO_2max_	60% VO_2max_	70% VO_2max_
Treadmill running		39.8 ± 3.0	4.7 ± 0.4	5.9 ± 0.5	7.0 ± 0.6
Resistance training		1RM (kg)	RM (kg)		
			60%	70%	85%
Upper body	Chest press Lat pull down Shoulder press Biceps curl Triceps extension	13.6 ± 3.6 26.1 ± 3.6 12.3 ± 4.3 15.8 ± 4.1 28.2 ± 7.0	8.2 ± 2.2 15.7 ± 2.1 7.4 ± 2.6 9.5 ± 2.5 16.9 ± 4.2	9.5 ± 2.5 18.3 ± 2.5 8.6 ± 3.0 11.1 ± 2.9 19.7 ± 4.9	10.9 ± 2.9 20.9 ± 2.8 9.8 ± 3.4 12.6 ± 3.3 22.5 ± 5.6
Core	Abdominal curl	17.9 ± 3.4	10.7 ± 2.0	12.5 ± 2.4	14.3 ± 2.7
Lower body	Leg press Leg extension Leg curl	41.6 ± 8.2 46.7 ± 15.6 24.3 ± 3.6	41.6 ± 8.2 46.7 ± 15.6 24.3 ± 3.6	29.1 ± 5.8 32.7 ± 10.9 17.0 ± 2.5	33.3 ± 6.6 37.3 ± 12.5 19.4 ± 2.9

Mean ± SD.

### Assessment

#### Basic anthropometric and bone mineral density measurements

Body composition was measured using a body composition analyzer (In-Body 720, Bio-Space Co., Seoul, Korea) in the same way before and after the 12-weeks exercise intervention period. The total bone mineral density (BMD) was measured by dual energy x-ray absorptiometry (DEXA) using a BMD analyzer (DPX-L, Lunar, WI, USA).

#### The maximum oxygen uptake measurement

The maximum oxygen uptake was measured using a treadmill ergometer (T150 DE, Cosmed, Rome, Italy), a respiratory gas analyzer (VO 2000 Metabolic Measurement system, Med Graphics, NY, USA) and a heart rate monitor (FS1, Polar Electro, Kemple, Finland). The modified Bruce protocol was applied, starting at 1.7 mph (10%), and increased every 3 minutes.

#### Blood collection and sample treatment, serum analysis

Ten milliliters of blood was collected from the brachial vein before and after the 12-week exercise intervention. Complementary DNA (cDNA) was synthesized by extracting total RNA from 5 mL of whole blood within 3 hours of blood collection, and experiments were analyzed to confirm the mRNA levels of RANKL signal transduction factors and cytokines. Also, the remaining 5 mL of blood was incubated at 5°C for about 30 minutes, then the serum was separated and stored at -80°C until analysis. Some samples were analyzed by an Enzyme-Linked Immunosorbent Assay (ELISA) of RANKL signaling factors and cytokines. The concentrations of OPG, RANKL, TNF-α, and IL-6 were analyzed using ELISA kits (Human OPG, RANKL, TNF-α, IL-6 ELISA kit, Fine Test, Wuhan, China). A special blood analysis company (SQLab, Seoul, Korea) analyzed the blood level of calcium, phosphorus, and magnesium. A limitation of this study is that the menstrual cycle was not considered during blood collection.

#### mRNA Expression of RANKL signal transduction factors and bone resorption cytokines

Total RNA was extracted from whole blood cells and mRNA analysis of OPG, RANK, RANKL signal transduction factors, TNF-α, IL-6, and other bone resorption cytokines, were measured by Real-time quantitative polymerase chain reaction (RT-PCR). To quantify the mRNA expression level of RANKL signaling factors and bone resorption cytokines, the relative amounts of glyceraldehyde 3-phosphate dehydrogenase (GAPDH) mRNA were analyzed. RNA extraction, cDNA synthesis, and real-time PCR were performed as described by Kim et al.^18^. Table 3 shows the primer sequences used in this study.

**Table 3:** Primer sequences used to measure mRNA levels of RANKL pathway members and cytokines

Gene		Primer sequences	
RANK	(+) (-)	5'-CTG ACT CTT CGA GAT CAT TT-3' 5'-CTA GGT CTT GGA CGT AAT AC-3'	64̊C
RANKL	(+) (-)	5'-TTA TAA CGA CCT GCA AGG TTA-3' 5'-TCC GGG CTT ACG ACG TAC CCT-3'	56̊C
OPG	(+) (-)	5'-AGT CCC TGG ACT GAA CTA AAG-3' 5'-ACC CCT GGT ATC ATT CAG GGC-3'	56̊C
TNF-α	(+) (-)	5'-GGG ACC TCT CTC TAA TCA-3' 5'-CTA CAA CAT GGG CTA CAG-3'	58̊C
IL-6	(+) (-)	5'-TGG ATT CAA TGA GGA GAC-3' 5'-TCT GGA GGT ACT CTA GGT ATA-3'	56̊C
GAPDH	(+) (-)	5'-AGA GAT GGC CAC GGC TGC TT-3' 5'-ATT TGC GGT GGA CGA TGG AG-3'	58̊C

RANK, Receptor activator of nuclear factor kappa B; RANKL, RANK ligand; OPG, Osteoprotegerin; TNF-α, Tumor necrosis factor-alpha; IL-6, Interleukin-6; GAPDH, Glyceraldehyde 3-phosphate dehydrogenase

### Statistical analysis

All data were calculated using SPSS for Windows (ver. 22.0) statistical program (SPSS Inc., IL, USA). The two-way repeated measure ANOVA method was performed for statistical analysis to analyze the difference (2 by 2) between the different time points (before and after exercise intervention) and the two groups (CON and EXE). If the primary effects between groups or times were statistically significant, the difference within each group was verified by conducting a t-test for independent samples, and within each time point was verified by conducting a t-test for paired samples. The statistical significance level (α) was set at less than 5% (p < 0.05).

## RESULTS

### Serum concentration and mRNA expression of RANKL signaling factors

After completing the 12-week intervention, there was no statistically significant group-by-time change of serum concentration or mRNA expression of RANKL signaling factors (Table 4). There was a statistically significant time effect on the serum mRNA levels of RANK (F = 17.611, p = .003) and RANKL (F = 8.13, p = .019), but t-test results showed a statistically significant decrease in both groups (p < 0.05, p < 0.001). Therefore, there was no significant difference according to the exercise program.

**Table 4:** Effects of a 12-week combined exercise on serum concentration and mRNA expression of RANKL signaling factors in female college students

Variables	Group	Before	After	Source	*F*	*P*
sRANKL (pg/ml)	CON EXE	277 ± 228 538 ± 440	313 ± 281 487 ± 389	group time g×t	1.773 0.024 1.521	0.216 0.881 0.249
sOPG (pg/ml)	CON EXE	43 ± 35 42 ± 33	32 ± 19 24 ± 13	group time g×t	0.112 3.596 0.559	0.746 0.09 0.474
sOPG/RANKL (ratio^b^)	CON EXE	0.19 ± 0.14 0.36 ± 0.64	0.15 ± 0.08 0.12 ± 0.12	group time g×t	0.459 1.709 1.415	0.515 0.224 0.265
RANK mRNA (ratio^b^)	CON EXE	31.21 ± 21.69 18.20 ± 16.08	2.39 ± 2.52^***^ 3.72 ± 2.10^*^	group time g×t	1.608 17.611 3.152	0.24 0.003 0.114
RANKL mRNA (ratio^b^)	CON EXE	11.48 ± 10.52 6.40 ± 4.75	2.77 ± 7.28^*^ 1.03 ± 0.56^**^	group time g×t	1.088 8.13 0.06	0.324 0.019 0.812
OPG mRNA (ratio^b^)	CON EXE	4.35 ± 3.55 3.50 ± 1.55	5.51 ± 4.80 2.94 ± 2.14	group time g×t	0.845 0.032 0.045	0.426 0.869 0.846
OPG/RANKL (ratio^b^)	CON EXE	0.75 ± 0.46 0.59 ± 0.53	11.32 ± 11.82 2.94 ± 1.33	group time g×t	3.136 4.899 2.082	0.175 0.114 0.245

Mean ± SD. CON, Control group; EXE, Exercise group; s, serum; RANK, Receptor activator of nuclear factor kappa B; RANKL, RANK ligand; OPG, Osteoprotegerin; g × t, group × time; b, ratio to the amount of GAPDH mRNA; ^*^:*p* < 0.05, ^*^^*^:*p* < 0.01, ^*^^*^^*^:*p* < 0.001 vs before.

### Serum concentration and mRNA expression of bone-resorbing cytokines

There was no significant group-by-time interaction for either TNF-α or IL-6 in serum concentration or mRNA expression, but there was a significant group effect of IL-6 mRNA expression (F = 37.98, p = 0.002) (Table 5). The t-test results showed that the expression of IL-6 mRNA in the EXE group was lower than that in the CON group after the 12-week intervention (p < 0.05).

**Table 5:** Effects of a 12-week combined exercise program on serum concentration and mRNA expression of TNF-α and IL-6 in female college students

Variables	Group	Before	After	Source	*F*	*P*
sTNF-α (pg/ml)	CON EXE	153 ± 139 114 ± 97	126 ± 135 154 ± 161	group time g×t	0.455 2.966 0.466	0.517 0.119 0.512
sIL-6 (pg/ml)	CON EXE	68 ± 73 38 ± 34	49 ± 71 60 ± 86	group time g×t	0.039 0.023 1.03	0.847 0.884 0.337
TNF-α mRNA (ratio^b^)	CON EXE	3.83 ± 4.96 1.76 ± 0.99	1.80 ± 3.06 1.51 ± 0.63	group time g×t	0.959 0.63 0.122	0.36 0.453 0.737
IL-6 mRNA (ratio^b^)	CON EXE	2.75 ± 2.46 2.84 ± 0.65	4.81 ± 1.78 2.43 ± 0.74^#^	group time g×t	37.98 0.725 2.345	0.002 0.433 0.186

Mean ± SD. CON, Control group; EXE, Exercise group; s, serum; TNF-α, Tumor necrosis factor-alpha; IL-6, Interleukin-6; g × t, group × time; b, ratio to the amount of GAPDH mRNA. #:*p* < 0.05, vs CON after.

### Bone mineral density and cardiorespiratory fitness

After completing the 12-week intervention, the total BMD results showed no statistically significant effect between the groups, the times, and the group-by-time interaction as analyzed by repeated measures ANOVA (Table 6). In addition, the maximum oxygen uptake did not show a statistically significant group-by-time interaction, but there was a significant effect in the group (F = 10.017, p = 0.011). t-test results showed that the cardiovascular fitness level of the CON group had significantly decreased after 12 weeks compared to the pre-treatment levels (p < 0.05). In addition, there was a significant time effect in the maximum heart rate (F = 8.678, p = 0.016), and the maximum heart rate of the CON group had decreased after the 12-week intervention (p < 0.05).

**Table 6:** Effects of a 12-week combined exercise program on bone mineral density and cardiovascular endurance in female college students

Variables	Group	Before	After	Source	*F*	*P*
Total BMD (g/cm^2^)	CON EXE	1.103 ± 0.051 1.109 ± 0.068	1.106 ± 0.053 1.108 ± 0.071	group time g×t	0.123 1.059 1.308	0.734 0.33 0.282
VO_2_ max (ml/kg/min)	CON EXE	39.84 ± 4.84 41.25 ± 4.80	37.29 ± 5.15^*^ 40.32 ± 3.90	group time g×t	1.172 10.017 2.19	0.307 0.011 0.172
HR max (BPM)	CON EXE	191.25 ± 6.94 195.90 ± 8.89	181.75 ± 13.19^*^ 194.80 ± 8.48	group time g×t	4.422 8.678 4.658	0.065 0.016 0.059
RER (ratio)	CON EXE	1.19 ± 0.07 1.22 ± 0.05	1.22 ± 0.10 1.18 ± 0.06	group time g×t	1.944 0.000 1.898	0.197 0.986 0.202

Mean ± SD. CON, Control group; EXE, Exercise group; g × t, group×time; ^*^:*p* < 0.05 vs before; BMD, Bone mineral density; HR, Heart rate; RER, Respiratory exchange ratio.

### Serum calcium metabolism markers

After completing the 12-week intervention, there were no statistically significant differences in serum calcium, phosphate, or magnesium levels (Table 7).

**Table 7:** Effects of a 12-week combined exercise program on serum calcium metabolism markers in female college students

Variables	Group	Before	After	Source	*F*	*P*
Calcium (mg/dl)	CON EXE	9.65 ± 0.23 9.66 ± 0.24	9.58 ± 0.33 9.68 ± 0.32	group time g×t	0.636 1.455 2.5	0.446 0.259 0.148
Phosphorus (mg/dl)	CON EXE	4.22 ± 0.65 3.86 ± 0.42	4.19 ± 0.46 3.93 ± 0.56	group time g×t	2.047 0.106 0.053	0.186 0.752 0.823
Mg (mg/dl)	CON EXE	2.15 ± 0.10 2.12 ± 0.12	2.12 ± 0.14 2.12 ± 0.14	group time g×t	0.041 1.122 1.841	0.844 0.317 0.208

Mean ± SD. CON, Control group; EXE, Exercise group; Mg, Magnesium; ALP, Total alkaline phosphatase; g × t, group × time.

## DISCUSSION

The binding of RANKL, which is secreted in osteoblasts, to its receptor RANK, which is secreted in osteoclasts, promotes osteoclast differentiation into cells with multiple nuclei and the expression of specific cytokines and hormones increases in osteocytes^11^. Therefore, RANK-RANKL signaling is a remarkable biological marker of bone resorption activation in osteocytes. OPG is known as the osteoclastogenesis inhibitory factor (OCIF), preventing the binding between RANK and RANKL^12^. In this study, to investigate the effect of prolonged exercise intervention on bone resorption, healthy college females performed a combined exercise program of 60 minutes per day, three times per week, for 12 weeks and their blood was drawn before and after the exercise intervention. Then, the serum concentration and mRNA expression of RANKL and OPG, and OPG/RANKL ratio from PBMCs were assayed and statistically analyzed. We examined these parameters from PBMCs since RANK is expressed in the circulating mononuclear cells and RANKL is expressed in T lymphocytes^22^ and B lymphocytes^23^. There was no statistically significant difference in group-by-time effect in all of the factors, indicating the absence of change following combined exercise, and this illustrates that prolonged exercise does not influence the bone resorption action at the cellular level.

A study by Pichler et al.^24^ using osteoporosis-induced rats reported that mechanical strain may activate RANKL signaling factors. Based on this previous study, it was expected that levels of the RANKL signaling factors would be changed in the exercise group of this study, but we observed no change in the serum concentration and mRNA expression. According to previous studies by Marques et al.^25^^,^^26^, serum OPG, RANKL, and OPG/RANKL ratios were observed after eight months of combined exercise intervention in elderly participants, and as a result, there was no significant change in all variables in both studies. That is why this study targeted healthy college females instead of elderly women and looked at mRNA expressions in PBMCs apart from serum variation alone, but significant changes were not found. However, in a study that assigned 27 middle-aged men into two groups of moderate-intensity (50-65% HRmax) and high-intensity (70-75% HRmax) instructed walking exercise for 10 weeks, five times per week^27^, the serum concentration of RANKL was reported to decrease only in the high-intensity group when the before and after levels were compared. This suggested that RANKL signaling factors may depend on exercise intensity. The present study conducted a combined exercise program of 60 minutes per day, three times per week, for a total of 12 weeks and instructed running on the treadmill with 50-70% VO_2_max intensity for aerobic exercise and resistance training with 60-85% RM while gradually increasing the intensity. Judging from the average of the 12 weeks, this only amounted to the aerobic exercise of 60% VO_2max_ intensity and resistance training of 72.5% RM intensity, so it can be regarded as intermediate intensity. Therefore, research examining higher exercise intensity should be conducted in the future.

In the meantime, an increase in inflammation has been reported to be related to reduced bone mineral density^28^^,^^29^. Especially, TNF-α and IL-6 are secreted from bone marrow cells, macrophages, PBMCs, etc., which reflect the bone marrow environment and are called bone-resorptive cytokines because of their involvement in the proliferation and activation of osteoclasts^30^. Therefore, we focused on the action where TNF-α, IL-1, and similar factors activate nuclear factor of activated T-cells (NFATC1) and cytoplasmic 1 and induce osteoclast progenitor cell differentiation of tartrate resistance acid phosphatase (TRAP), cathepsin K, and DC-STAMP. Furthermore, we studied the bone-resorptive inhibitory action of cytokines including TNF-α, IL-1, and IL-6 according to this mechanism and various exercise interventions^14^^-^^16^. Particularly, TNF-α and IL-6 are known to promote the differentiation of osteoclastic progenitors into osteoclasts through passing prostaglandin E2 (PGE2) in bone tissue and increasing the secretion of RANKL on the cellular surface of osteoblasts^31^. In addition, OPG synthesis is known to be induced and stimulated by TNF-ɑ, IL-1, steroid hormones, and other factors, whereas parathyroid hormone and PGE2 suppress the production of OPG^32^. Steeve et al. [33] reported, based on the results of their in vitro studies, that an increase of TNF-ɑ, IL-1, and 1L-6 promotes the production of RANKL and OPG, which are related to osteoclast differentiation and proliferation. However, our present study showed no interaction between group and time on the serum TNF-α and IL-6 concentration as well as their mRNA expression. There was a significant group effect of IL-6 mRNA expression, indicating low IL-6 mRNA expression in the EXE group.

Previous studies reported changes in TNF-ɑ and IL-6 after a long-term exercise intervention. In a study where 47 elderly male and female participants performed combined exercise for 32 weeks, it was reported that the serum level of IL-6 decreased after the exercise program^26^. Similarly, another study that examined the effects of aerobic exercise three times per day for 24 weeks in menopausal women reported decreases in serum TNF-ɑ and IL-6 levels upon combined exercise and supplement intake, demonstrating that treatment is effective for decreasing inflammation and preventing reduced bone density in menopausal women^34^. Thus, even if long-term exercise treatment does not influence RANKL signaling factors, it generally reduces levels of bone-resorptive cytokines like TNF-ɑ and IL-6. In the present study, there were no significant differences in levels of TNF-α and IL-6 and RANKL signaling factors in serum, and only IL-6 mRNA expression was relatively low; we consequently confirmed that it is imperative for future studies to include a variety of exercise interventions. It is possible that 12 weeks of exercise training might be not enough to induce a detectable change.

The results of the present study also point towards the need for further studies. After 12 weeks of combined exercise, the maximum oxygen intake and maximum heart rate of the exercise group did not increase, and bone density and calcium metabolism did not improve either. That is, the intensity of the combined exercise performed in this study was not intense or voluminous enough to improve cardiorespiratory fitness, and therefore, it did not largely impact RANKL signaling factors or improve whole-body bone density. Generally, there is no change in bone density in conditions where the intensity or the volume of exercise is low, such as aqua aerobics twice per week^35^, but progressive resistance strength training or combined exercise programs of sufficient intensity is known to improve bone density^36^. Therefore, it is suggested that physical fitness is enhanced through a sufficient exercise load, and future studies should examine the relationship between changes in levels of RANKL signaling factors and bone density to evaluate the effect of exercise.

## CONCLUSION

In this study, we conducted a combined exercise program three times per week for 12 weeks in healthy female college students. As a result, exercise did not improve participants’ cardiorespiratory fitness. In addition, although there was a significant difference in IL-6 mRNA expression level, levels of the RANKL signaling factors did not change, and the whole-body BMD did not change compared with the control group. In conclusion, we demonstrated that without the effect of sufficient exercise to improve cardiorespiratory fitness, the effect on RANKL/RANK/OPG signaling and bone-resorptive cytokines will also be limited.
